# An Urdu speech *corpus* for emotion recognition

**DOI:** 10.7717/peerj-cs.954

**Published:** 2022-05-09

**Authors:** Awais Asghar, Sarmad Sohaib, Saman Iftikhar, Muhammad Shafi, Kiran Fatima

**Affiliations:** 1 Sino-Pak Center for Artificial Intelligence, Pak-Austria Fachhochschule: Institute of Applied Sciences and Technology, Haripur, Pakistan; 2 Department of Electrical Engineering, University of Engineering and Technology, Taxila, Punjab, Pakistan; 3 Department of Electrical and Electronic Engineering, University of Jeddah, Jeddah, Saudi Arabia; 4 Faculty of Computer Studies, Arab Open University, Riyadh, Saudi Arabia; 5 Department of Computing, School of Electrical Engineering and Computer Science, National University of Science and Technology, Islamabad, Pakistan; 6 Faculty of Computing and Information Technology, Sohar University, Sohar, Oman; 7 TAFE, New South Wales, Australia

**Keywords:** Human computer interaction, Linear prediction coefficient (LPC), Mel frequency capstrum coefficient (MFCC), Speech descriptors, Machine learning algorithms, Urdu, Emotion recognition, Human behavior analysis

## Abstract

Emotion recognition from acoustic signals plays a vital role in the field of audio and speech processing. Speech interfaces offer humans an informal and comfortable means to communicate with machines. Emotion recognition from speech signals has a variety of applications in the area of human computer interaction (HCI) and human behavior analysis. In this work, we develop the first emotional speech database of the Urdu language. We also develop the system to classify five different emotions: sadness, happiness, neutral, disgust, and anger using different machine learning algorithms. The Mel Frequency Cepstrum Coefficient (MFCC), Linear Prediction Coefficient (LPC), energy, spectral flux, spectral centroid, spectral roll-off, and zero-crossing were used as speech descriptors. The classification tests were performed on the emotional speech *corpus* collected from 20 different subjects. To evaluate the quality of speech emotions, subjective listing tests were conducted. The recognition of correctly classified emotions in the complete Urdu emotional speech *corpus* was 66.5% with K-nearest neighbors. It was found that the disgust emotion has a lower recognition rate as compared to the other emotions. Removing the disgust emotion significantly improves the performance of the classifier to 76.5%.

## Introduction

**Figure 1:**
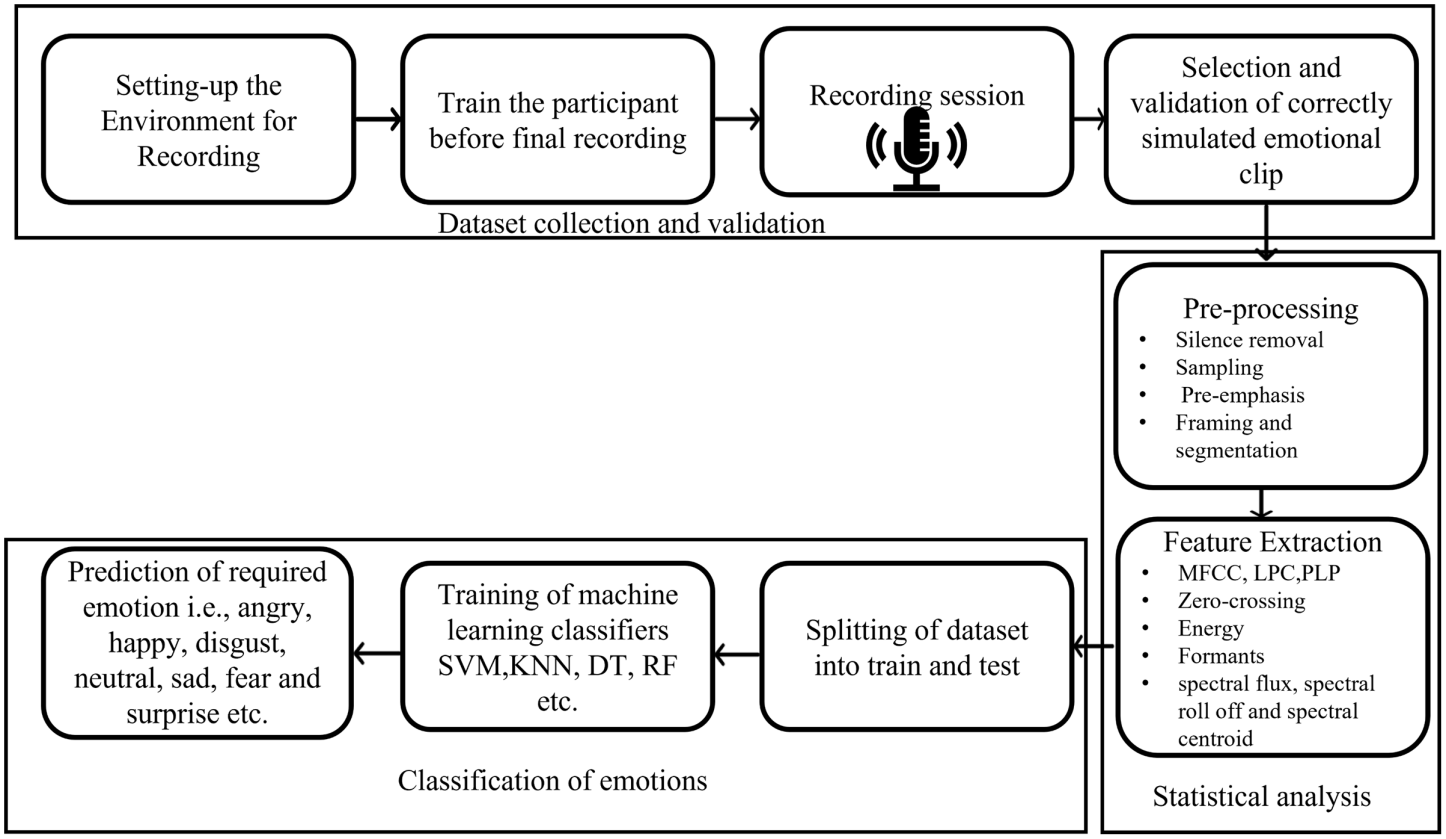
Emotion recognition pipeline.

Emotion recognition is a vital aspect towards complete human-machine interaction since effective communications of information is fundamental to human-machine interaction. Emotion recognition is also a vital part of automatic human behavior analysis such as assessing candidates’ suitability for a job, assessing emotional intelligence, and lie detection, *etc*. There are many ways in which machines can recognize emotions such as face recognition, gestures, eye movements, body language, and electrocardiogram (ECG) signals (Soleymani et al., 2016). Among all these, speech is an easy and effective form of interaction. Hence, the literature in emotion detection research is focused on the interpretation of emotions from human speech (Dahake, Shaw & Malathi, 2016). There are several applications of emotional understanding such as E-learning where the tutor can change the presentation style when a learner is feeling uninterested, angry, or interested. Similarly, in medical sciences, virtual assessment of the patients’ health is possible by listening to his/her voice. In the robot-human communication, the robots can be trained to communicate with human-based emotional states. The cellular services, multimedia devices and call centers have vast area of application related to emotion recognition where devices can detect the human behavior (frustration and annoyance etc.) of end user and react accordingly. Usually, the emotion recognition from the speech is performed by collecting datasets (training, testing and validation), performing statistical analysis (extraction of the features that are associated to the different emotional states), and to classify the emotions from the acoustic signals, as illustrated in Fig. 1 (Yadav & Aggarwal, 2015).

Extensive literature is available in the field of human emotions recognition for different languages such as English, French, German, and Malayalam in the last few years (Ververidis & Kotropoulos, 2003). For these languages, the developed emotional speech datasets comprises the collection, careful annotation, noise filtering, and validation of speech samples. However, such databases need to be developed for other global languages. The Urdu language has more than 11 million speakers worldwide as a native language and 105 million second-language speakers in the world (BBC, 2022). However, speech emotion recognition (SER) from the Urdu language needs further research (Qasim et al., 2016; Kaminska, Sapinski & Anbarjafari, 2017) and significant improvements such as noise filtering, careful annotation and validation of samples in the development of the Urdu language emotional dataset. Owing to this lack of consideration in Urdu language dataset collection, Urdu emotional speech database with noise filtering, careful annotation, and sample validation features is realized in this study. The emotion recognition performance is predominantly affected by the pre-processing, feature extraction, and algorithms used to classify the speech into various emotions. In this study, K-nearest neighbour (k-NN), Random Forest (RF), and multiclass Support Vector Machine (SVM) with the linear kernel are used to validate the efficiency of the feature sets.

The remainder of this article is organized as follows. “Background and Related Work” describes the related work and background of research. “Dataset Collection” provides an overview of Urdu emotional speech *corpus* collection, assignments of labels, and Urdu utterances selected for the recording. “Pre-Processing” explores the pre-processing. “Feature Extraction” provides details of feature extraction, and ML algorithms. “Results and Discussions” presents the classification results. Finally, “Conclusion” concludes the paper with future directions.

## Background and Related Work

In the field of natural language processing (NLP) and automatic speech recognition (ASR), several speech corpora have been developed for various languages (Douglas-Cowie et al., 2003; Dimitrios Ververidis, 2019). Many successful proposals have been proposed in the emotion classification for resource rich languages such as Italian (Giovannella et al., 2009), Polish (Staroniewicz & Majewski, 2009), German (Grimm, Kroschel & Narayanan, 2008), English (Livingstone & Russo, 2018), and French (Gournay, Lahaie & Lefebvre, 2018). However, emotion recognition in the Urdu language is still a target research area and there is a sufficient opportunity for the improvement. Due to the insufficiency of the emotion recognition techniques for the Urdu language, emotion recognition systems for other languages are summarised below, followed by such systems for the Urdu language.

Livingstone & Russo (2018) and Zhang, Provost & Essi (2016) presented a multimodal English language emotional speech and song *corpus* in Livingstone & Russo (2018), Zhang, Provost & Essi (2016). The dataset is collected from 24 professional actors by simulating two neutral statements, that is, “Dogs are sitting behind the door” and “Kids are talking by the door”. Seven emotions are selected for the speech whereas five for the song, respectively. Every emotion is simulated with two levels of intensity that is strong and neutral. To validate the dataset, 247 untrained individual opinions are taken on each emotion. Kaminska, Sapinski & Anbarjafari (2017) developed an emotion recognition framework for the Polish language, where the dataset is recorded in two different forms of emotional speech that is spontaneous and acted speech. Spontaneous speech samples are collected from live TV shows and programs such as news and reality shows. The acted speech samples are recorded from eight native speakers of both genders (four males and four females) where they uttered 240 sentences in six different emotions. The validation of the dataset is endorsed by the subjective listening test. An accuracy of 72% is achieved in emotion recognition. Statistical analysis is also performed to validate the *corpus*. A pool of the features including Perceptual Linear Prediction (PLP), Bark Frequency Cepstral Coefficient (BFCC), and Human Factor Cepstral Coefficients (HFCC) is used to classify the emotions. The achieved accuracy of this experiment for natural and acted speech is 81% and 60% respectively. Lyakso et al. (2015) developed the first emotional speech *corpus* of children in the Russian language and named as the EmoChildRu. It was comprised of audio samples of 120 children simulated in three different emotions including the comfort, discomfort, and neutral. The basic emotions of anger, sadness, and fear are expressed as discomfort. Leila et al. (2019) achieved an accuracy of 83% in recognition of seven basic emotions on the German EmoDB database after applying feature selection and speaker normalization techniques. The Mel Frequency Capstrum Cofficient (MFCC) and Modulation Spectral Features (MSFs) methods were used for feature extraction. Kumar & Iqbal (2019) and Khalil et al. (2019) discussed different classifiers such as k-NN, SVM, convolutional neural networks (CNN), recurrent neural networks (RNN), and long short-term memory (LSTM) and some feature extraction techniques in Kumar & Iqbal (2019), Khalil et al. (2019) and Zhao, Mao & Chen (2019), respectively. Pengcheng & Zhao (2019) proposed an emotion recognition system for the Chinese language, where denoising auto-encoder and sparse autoencoder are used for feature extraction whereas the wavelet kernel sparse SVM classifier is used for the classification. Tripathi & Beigi (2018) have used RNN with three hidden layers to recognize emotion for the IEMOCAP database with an accuracy of 71.04%. This study used only four emotions that is happiness, sadness, neutral, and anger. Tang, Zeng & Li (2018) recognized seven basic emotions from the *corpus* named as emotional sensitivity assistance system for people with disabilities (EmotAsS) (Simone et al., 2017) and achieved an accuracy of 45.12% with RNN, CNN and ResNet. Sarma et al. (2018) and Eskimez, Duan & Heinzelman (2018) used the IEMOCAP dataset for sentiments recognition, where classification is carried out using the LSTM and CNN. An accuracy of 70.06% and 47% is achieved for LSTM and CNN, respectively. Latif et al. (2018) presented a cross-lingual recognition system: Urdu *vs* Western language. A recognition accuracy of 83.04% was achieved for the Urdu dataset when other languages are used in training set on four basic emotions. SVM, logistics regression, and random forest are used for classification. Panagiotis et al. (2017) proposed a system with RNN and ResNet that gives recognition rates of 78.7% on the French language based remote collaborative and affective (RECOLA) dataset. The details of the RECOLA are explained by Fabien et al. (2013). Mao et al. (2017) introduced an Emotion-discriminative and Domain-invariant Feature Learning Method (EDFLM) in Mao et al. (2017). It provided a good emotion recognition rate on the INTERSPEECH 2009 challenge and the Emo-DB database. Fayek, Lech & Cavedon (2017) and Mirsamadi, Barsoum & Zhang (2017) both use the IEMOCAP dataset with RNN and CNN obtained 64.78% and 63.5% of accuracy, respectively. Mirsamadi, Barsoum & Zhang (2017) used both Low-Level Descriptors (LLDs) and High-Level Statistical Functions (HSFs) as input to SVM in order to differentiate emotions. Rajisha, Sunija & Riyas (2016) performed analysis on the Malayalam language to differentiate different sentiments. MFCC, energy, and pitch are used for features extraction. The four basic emotions (happiness, sadness, neutral, and anger) are classified by SVM and artificial neural network (ANN). Yadav & Aggarwal (2015) achieved an 85% accuracy to recognize four emotions with ANN. Sinith et al. (2015) tested the SVM with two classification strategies that is one against one, and one against all in Sinith et al. (2015). The SVM gives a higher performance on Berlin emotional database as compared to Malayalam emotional database with a feature set of MFCC, energy, and pitch. Abbas, Khan & Bashir (2015) performed a classification of emotions for Urdu language (Abbas, Khan & Bashir, 2015) where J48 and Decision tree are tested, achieving an accuracy of 48% with four basic emotions. Fayek, Lech & Cavedon (2015) achieved an emotions recognition rate on eINTERFACE and SAVEE database in Fayek, Lech & Cavedon (2015) which was 60.53% and 59.7%, respectively. The Polish language emotion speech dataset obtained 70% accuracy with k-NN.

Table 1 presents a summary of the emotion recognition techniques from the literature. Rauf et al. (2015) proposed a speaker-independent Urdu language speech recognition system where the dataset comprises the utterances for district names of Pakistan. A total of 139 district names are recognized in major Urdu language accents such as Punjabi, Sindhi, Balouchi, and Pashto. Ali et al. (2013) presented an Emotions-Pak *corpus*, where only one utterance “In seven hours it will happen” is recorded in Urdu and other provincial languages of Pakistan. In this *corpus*, four emotions are obtained in a given sentence. To evaluate the performance of recorded emotions, results from the prosodic feature set and subjective listening were compared. Andleeb, Haider & Abbas (2017) performed the classification of the special and normal children’s speech emotions in Urdu language. A total of 11 different feature extraction techniques including MFCC, Linear Prediction Coefficient (LPC), and PLP are used to classify the special and normal children’s speech. The dataset was recorded using 200 special and 200 normal children in four different emotions on the selected utterance “I have to play” in Urdu. Abbas, Zehra & Arif (2013) presented a system that recognized the emotions in the provisional languages of Pakistan, where only one utterance was simulated in Pakistani languages for four basic emotions. The achieved accuracy was 75% where Multi-layer Perceptron (MLP), and Naive Bayes were used as classifiers.

**Table 1:** Summary of literature on emotion recognition from different languages.

Papers with year	Dataset used	Emotions recognized	Technique used	Achieved accuracy
Leila et al. (2019)	Berlin EmoDB	Anger, disgust, fear, joy, neutral, surprise and sadness	SVM and multivariate linear regression (MLR)	83%
Kumar & Iqbal (2019)	EmoDB dataset	Neutral anger and sad	Deep belief network (DBN) and Stacked encoder	65%
Pengcheng & Zhao (2019)	Chinese emotional speech dataset	Anger, scared, happiness, sadness, neutral and surprise	Wavelet-kernel sparse SVM	80.95%
Tripathi & Beigi (2018)	IEMOCAP dataset	Anger, happiness, sadness, and neutral	RNN with 3 layers	71.04%
Tang, Zeng & Li (2018)	EmotAss dataset	Anger, happiness, neutral and sadness	CNN and RNN with ResNEt	45.12%
Sarma et al. (2018)	IEMOCAP dataset	Anger, happiness, neutral, sadness, surprise, fear and disgust	LSTM	70.6%
Eskimez, Duan & Heinzelman (2018)	IEMOCAP dataset	Neutral, sadness, frustration and anger	CNN	47%
Latif et al. (2018)	Urdu language emotional speech dataset	Anger, happiness, neutral and sadness	SVM, logistic regression and random Forest	83.4%
Panagiotis et al. (2017)	Spontaneous emotional RECOLA and AVEC dataset	Happiness, sadness, anger and neutral	CNN and ResNEt	78.7%
Mao et al. (2017)	INTERSPEECH 2009, ABC and EmoDB	Happiness, sadness, neutral, fear, surprise, disgust and anger	Emotion discriminative and domain invariant feature learning method (EDFLM)	65.62%
Fayek, Lech & Cavedon (2017)	IEMOCAP dataset	Neutral, happiness, sadness, anger and silence	RNN and CNN	64.78%
Kaminska, Sapinski & Anbarjafari (2017)	Acted and spontaneous Polish language dataset	Sadness, happiness, anger, neutral, joy, fear, and surprise	SVM and k-NN	81%
Mirsamadi, Barsoum & Zhang (2017)	IEMOCAP dataset	Neutral, anger, sadness, and happiness	Recurrent Neural Network RNN and SVM	63.5%
Zhang et al. (2017)	Chinese emotional speech dataset	Sadness, joy, anger, neutral fear, and surprise	SVM and Deep learning	84.54%
Zhu et al. (2017)	Chinese emotional speech dataset	Sadness, joy, anger, neutral fear, and surprise	Combination of SVM and Deep learning	95.8%
Rajisha, Sunija & Riyas (2016)	Malayalam language emotional speech dataset	Neutral, anger, happiness and sad	ANN and SVM	78.2%
Yadav & Aggarwal (2015)	English emotion speech dataset	Sadness, happiness, anger and neutral	Artificial Neural Network ANN	85%
Qasim et al. (2016)	District name of Pakistan dataset		SVM and GMM	71%
Sinith et al. (2015)	SAVEE and Malayalam emotional speech dataset	Anger, happiness, neutral and sadness	Support vector machine	75%
Abbas, Khan & Bashir (2015)	Urdu language emotional speech dataset	Anger, sadness, happiness and neutral	Decision tree and J48	40%
Fayek, Lech & Cavedon (2015)	ENTERFACE and SAVEE dataset	Boredom, disgust, sadness, joy, anger and neutral	Deep neural network DNN	60.53%
Abbas, Zehra & Arif (2013)	Provisional language of Pakistan emotional speech dataset	Comfort, happiness, sadness and neutral	Multilayer perceptron (MLP), Naïve Bayes and SMO	75%
Kami´nska & Pelikant (2012)	Polish emotional speech dataset	Sadness, happiness, anger and neutral	k-NN	70%

## Dataset Collection

**Figure 2:**
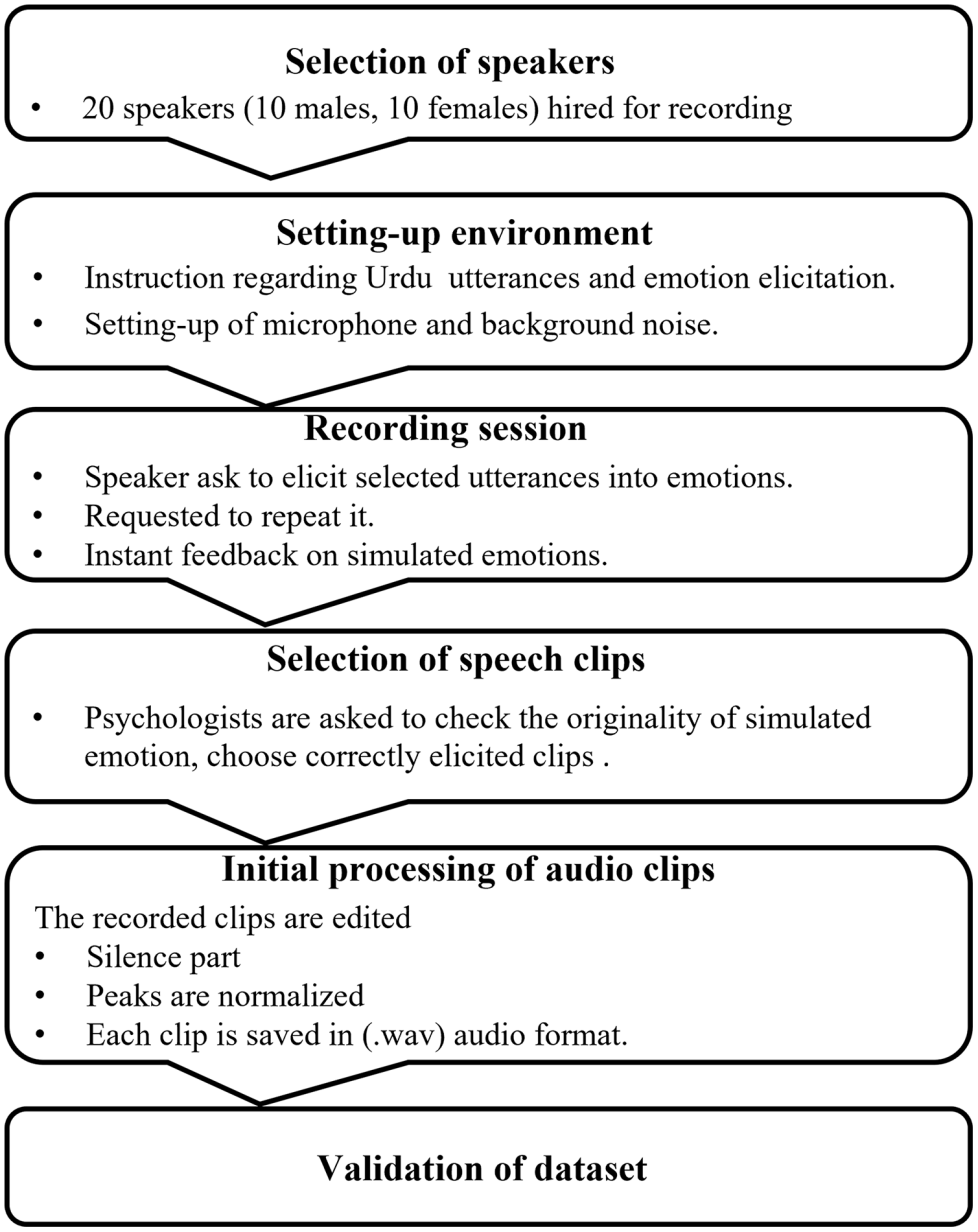
Flow chart of Urdu emotional speech dataset creation and validation.

Our emotional speech *corpus* comprises 2,500 emotion samples of Urdu speech. There are 20 speakers of both genders (10 males and 10 females) aging between 20 to 40 years. Each speaker utters five times. Every time a speaker utters five different Urdu utterances in five different emotions such as happy, sad, angry, disgust, and neutral. The selected utterances are everyday human-human interaction utterances and easy to understand in all five emotions. The utterances were recorded in the university lab using the Blue Yeti desktop microphone as recording equipment. After collection, the recorded emotional speech utterances were listened by a psychologist and a group of students (10–15) to verify the originality of simulated emotions. The speech utterances which were repeatedly mismatched with the assigned labels were discarded from the emotional *corpus*. A large number of samples were discarded from the disgust emotion which was also highlighted in the Results and Discussion sections. For this reason, the samples per emotion were not balance. The fully filtered emotional speech dataset was then fed to the emotion recognition system. The complete process of the emotional speech dataset is outlined in Fig. 2.

### Description of audio speech clips

The Urdu emotional speech dataset contains a total of 2,500 audio clips that was simulated by 20 speakers of both genders. Each speaker uttered 125 emotional speech clips that include five emotional states that were angry, happy, neutral, disgust, and sad on five commonly used Urdu language utterances. The full constructed data recording includes the number of clips per speaker = angry (5) × utterances (5) × repetition (5) = 125; for 20 speakers, the total number of audio clips became 125 × 20 = 2,500. In the validation stage, 200 samples, which were not correctly uttered, were filtered out. The distribution of remaining 2,300 audio clips/emotional speech samples is provided in Table 2.

**Table 2:** Number of emotions per sample.

Emotion	Number of samples
Angry	500
Disgust	400
Happy	500
Neutral	450
Sad	450

### Recording environment

The utterances were recorded in a noise-free lab room in absence of the background noise to achieve good quality. The speakers were asked to sit in front of a microphone, and they may move their bodies freely to express a particular emotion. Further, the speakers were asked to speak in the direction of a microphone to capture the full intensity of voice. The distance between the subject and recording equipment is kept at 25 cm.

### Acted or real emotion

A fully developed emotion appears occasionally in the real-life. From the real-life speech samples, it is almost impossible to differentiate between some basic emotions (Burkhardt et al., 2005). Hence the literature prefers the acted emotions. There are a few factors to be considered while collecting acted speech. (I): All speakers should act the same verbal content in order to allow the comparability across emotions and speakers. (II): The quality of the recorded voice assumed to be good enough, minimizing background noise; otherwise spectral measurements would not be possible. (III) a reasonable number of speakers should perform all emotions to obtain generalization over the target emotions.

### Choice of emotions and speakers

To compare the selection of emotions with early research (Yadav & Aggarwal, 2015; Giovannella et al., 2009; Grimm, Kroschel & Narayanan, 2008), the same emotions were used, such as: happy, sad, angry, disgust, and neutral. These emotions attract more attention and used in the human daily life. These selected emotions are easy to understand by the speakers as well as the listeners. It is important to note that we have not involved trained actors in performing emotional expression. All the speakers were students and faculty members of the department. However, the speakers were aware and trained before the actual recording of the emotions.

### Text material

The utterances used were easy to understand in the emotions, that is, there were no emotional biases involved. The literature suggested two types of text materials that can ensure such requirements (Costantini et al., 2014), (I): the text material that was emotionally neutral, and (II): normal sentences which are used in everyday life. In the preparation of the database, priority was given to the neutrality of speech material, and thus everyday sentences were used as test utterances. Five sentences were chosen which could easily be interpretable in the above-mentioned emotions. These sentences are given in Table 3.

**Table 3:** Chosen Urdu language utterances with English translation.

Sentences in Urdu	English translation
Pakistan kesa hai?	How is Pakistan?
qareeb tareen hospital kahan hai?	Where is the nearest hospital?
kapre fridg pr parey hein	The cloths are lying on the fridge.
tum kahan gaye they?	Where did you go?
kahan ho ajj kal?	Where are you nowadays?

### Recording of data

There was only one session of recording per day with three speakers. All the recordings were completed under the supervision of psychologist and experts, and their opinions on the emotion were also recorded. The collected speech samples were normalized and stored in “.wav” format with sampling frequency 44.1 kHz, and 16 bits per sample. A Blue Yeti desktop microphone was used to record the speech samples. The utterances were recorded in a noise-free lab room in absence of the background noise to achieve the good quality (Gournay, Lahaie & Lefebvre, 2018).

### Database validation

Based on the opinions of experts and psychologists during the collection stage, the utterances were extracted and initially classified into one of the five discrete emotion categories including happiness, sadness, anger, disgust, and neutral state. A psychologist was asked to listen carefully the randomly presented audio files and indicate which of the emotion is available in the presented files. The psychologist was not allowed to go back to previously presented emotion. Another labelling exercise was carried out where 10 to 15 students were included in the tests. Every student was presented with the acted emotions (.wav audio files) to make a decision about the simulated emotions and check the performance of speakers. Therefore, the speech samples which repeatedly mismatched with the labels were discarded from the emotional *corpus*. The fully filtered emotional speech dataset was then fed to the developed emotion recognition system. The recognition rate of each emotion is shown in Table 4.

**Table 4:** Recognition rate of each emotion during validation process.

Emotion	Recognition rate
Angry	96%
Sadness	94%
Neutral	92%
Happy	80%
Disgust	76%

## Pre-Processing

**Figure 3:**
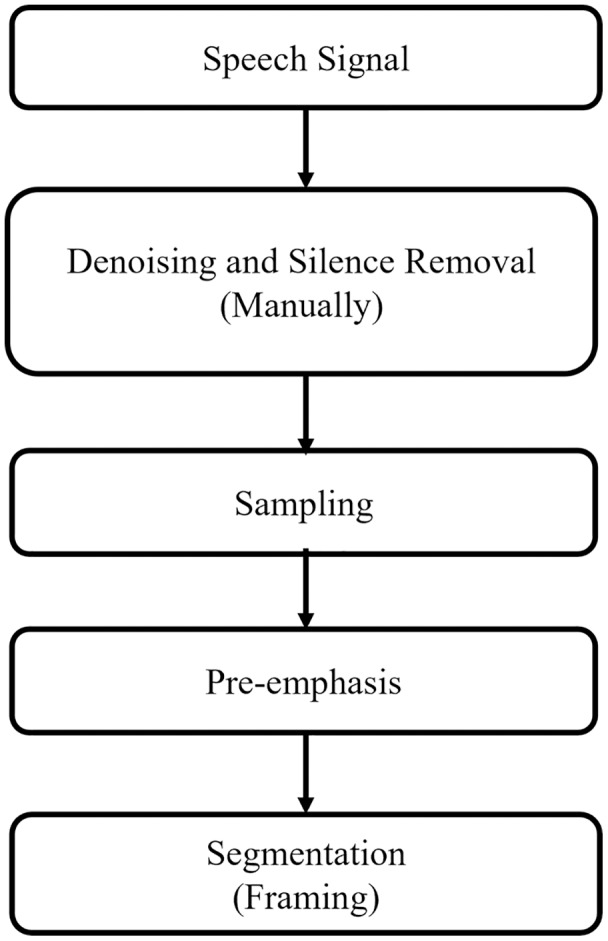
Pre-processing flow of speech signal.

In the emotion recognition system, there can be silence parts and background noise in the spoken utterances. Therefore, the emotional speech signals recordings from the microphone are first pre-processed and made them suitable and noise-free for feature extraction stage. In this study, silence parts and background noise are removed manually. Figure 3 demonstrates the pre-processing steps which are discussed in the subsections.

### Pre-emphasis

The high-frequencies were suppressed during the sound production by humans. Therefore, pre-emphasis was applied on the sampled signal to increase the magnitude of higher frequencies, thereby improving the overall signal-to-noise ratio (SNR). The pre-emphasis was implemented as a first order Finite Impulse Response (FIR) filter which is defined as:

(1) $$y\left( n \right) = x\left( n \right) - a\; x\left( {n - 1} \right),$$

where $y\left( n \right)$ is the emphasized signal, $x\left( n \right)$ is sampled signal and *a* is the pre-emphasis coefficient, with value raging from 0.9 to 1.0.

### Framing

Speech signal is non-stationary by nature and the spectral analysis usually considers the stationary signals. Therefore, framing was used to convert the non-stationary speech signals into stationary signals. During the framing, the speech signal was divided into a series of the overlapping frames. The frame length was 20 to 30 ms with an overlap of 1*/*3 of the frame size. Overlapping was used to avoid loss of data due to aliasing.

### Hamming window

The sudden change at the onset and offset of frame causes loss of important information. Therefore, Hamming windowing function was applied to all frames. If w(n) is the Hamming window function and y(n) is the input signal frame, then output z(n) is given by equation as:

(2) $$Z\left( n \right) = y\left( n \right)w\left( n \right),$$

where

(3) $$w\left( n \right) = 0.54 - 0.46\; {\rm cos}\left( {\displaystyle{{2\pi } \over {N - 1}}} \right),$$

N is number of samples in a frame and z(n) is a final pre-processed signal.

## Feature Extraction

After all pre-processing, the signal is appropriate for feature extraction. Various statistical values were used in our model to discriminate emotion classes. These statistical values are in the form of vectors known as feature vectors. These feature vectors provide a higher level of representations of audio samples. The extracted features in this study are explained below.

### Spectral flux

It is a one-dimensional feature vector against one audio sample. It is a measure of how rapidly the power spectrum of a speech signal varies and is calculated by comparing the power spectrum of two successive frames and computed as the squared difference between the standardized magnitudes of spectra of two consecutive short-term windows and is given by Alías, Socoró & Sevillano (2016)

(4) $${\rm Spectral\; Flux} = {\left( {\left| {z\left( n \right)} \right| - \left| {z\left( {n - 1} \right)} \right|} \right)^2}.\;$$

It is also known as the Euclidean distance among the two standardized spectra.

### Spectral centroid

The spectral centroid shows where the centre of gravity of the spectrum of the audio signal is located (Kamarudin et al., 2014). It is obtained by taking a weighted average of the frequency components present in the signal. The weighted average is determined by taking Fourier transform of frequencies and their magnitude as weights and calculated as:

(5) $${\rm Spectral\; Centroid} = \displaystyle{{\mathop \sum \limits_{{\rm n} = 0}^{{\rm N} - 1} {\rm n\; }{{\rm z}_{\rm t}}\left( {\rm n} \right)} \over {\mathop \sum \limits_{{\rm n} = 0}^{{\rm N} - 1} {\rm \; }{{\rm z}_{\rm t}}\left( {\rm n} \right)}},$$

where ${Z_t}\left( n \right)$ is the magnitude of Fourier transform at frame $\; t$ and frequency bin $\; n$.

### Spectral roll off

Spectral roll-off is a feature that is defined as the frequency under which 85% of the signal’s spectral energy is accumulated. This measurement gives the centre of mass of energy (higher frequencies) in the spectrum (Kaur & Kumar, 2017).

### Zero crossing

Zero crossing is a method to classify the voice and non-voice parts of the signal. It is the rate at which speech signals passes through zero level (Toledo-Pérez, Rodríguez-Reséndiz & Gómez-Loenzo, 2020). Zero crossing for the signal can be calculated as

(6) $${\rm Zero} - {\rm crossing} = \displaystyle{1 \over {\rm N}}\mathop \sum \limits_{{\rm n} = 0}^{\rm N} \left| {{\rm z}\left( {\rm n} \right)} \right| - \left| {{\rm z}\left( {{\rm n} - 1} \right)} \right|.$$

### Energy

Energy is a very basic and fundamental feature in signal processing (Li & Sun, 2008). Energy of speech signal is referred to an intensity of a signal and is calculated as

(7) $${\rm Energy} = \displaystyle{1 \over {\rm N}}\mathop \sum \limits_{{\rm n} = 0}^{\rm N} \left| {z{{\left( n \right)}^2}} \right|,$$

For example, energy of the happy and angry is different from sad and neutral.

### Linear prediction coefficient

The LPC model describes the vocal tract of the humans. In LPC, each sample of the speech signal is expressed as a linear combination of the earlier samples. These coefficients are highly effective representation of the speech signal (Alim & Rashid, 2018; Dave, 2013). In this analysis, each speech sample is represented by a weighted sum of past speech samples plus an appropriate excitation. The corresponding expression for the LPC model is given as:

(8) $${S_n} = \mathop \sum \limits_{k - 1}^p a\left( k \right)\; z\left( {n - k} \right) + e\left( n \right),$$

where *p* is the order of LPC, $a\left( k \right)$ is the *k*th coefficient of LPC vector, $z\left( {n - k} \right)$ is the ${n_{th}}$ speech sample and $e\left( n \right)$ is the prediction error. The coefficients $a\left( k \right)$ are computed by minimizing the sum of squared differences between the actual speech samples and the linearly predicted ones.

### Mel frequency capstrum coefficient

MFCC are the commonly used features in speech recognition systems. It is a short-term power spectrum of an audio signal, which is based on the inverse fast Fourier transform (IFFT) of a log power spectrum on a nonlinear Mel scale of frequency. The Mel scale is a perceived pitch or frequency that is heard by the listener to be equal in distance from one another. Human ear can easily understand the difference between pitch changes at low frequency as compared to high frequency. The incorporation of this scale makes our feature vector more closely related to the human hearing system (Alim & Rashid, 2018; Dave, 2013). Mel scale frequency can be expressed as:

(9) $${f_{mel}} = 1125\ln \left( {1 + \displaystyle{f \over {700}}} \right),$$

where *f* is a linear frequency and ${f_{mel}}$ is perceived frequency of speech signal. To move back to linear frequency scale from Mel scale perceived frequency we use

(10) $$f = 700\left( {{e^{{{{f_{mel}}} \over {1125}}}} - 1} \right)$$

MFCC is implemented using the following steps.

1. Segmented the time-domain speech signal.
2. For each segment, the periodogram estimate of discrete Fourier transformed (DFT) segments is calculated.
3. Applied the Mel scale filter bank on power spectrum, and sum-up the energy for each filter bank.
4. Take the log of Mel scaled energies.
5. Applied the discrete cosine transform (DCT) on a log Mel scaled energies.
6. Keep the first 13 DCT coefficients.

For one audio sample, the total feature vector size is 1 × 64 as summarized in the Table 5.

**Table 5:** Feature dimensions.

Features name	Features dimensions
MFCC	13
Mean of MFCC	13
Standard deviation of MFCC	13
LPC	10
Mean of LPC	10
Spectral flux	01
Spectral centroid	01
Spectral rolloff	01
Zero crossing	01
Energy	01
Total feature vector	64

## Results and Discussions

**Figure 4:**
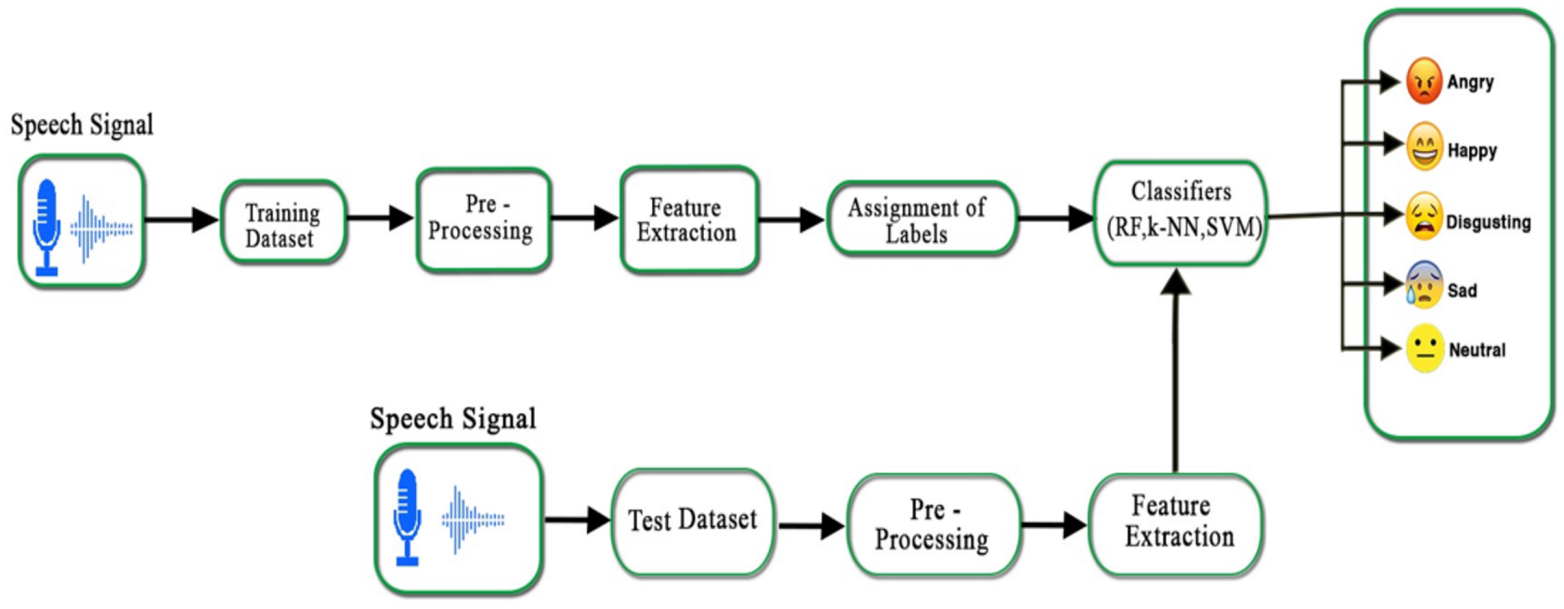
Proposed emotion recognition system for Urdu speech signals.

There are five main blocks in a speech emotion recognition system, that is, emotional speech input, pre-processing, feature extraction, assignment of labels, and classification of the emotions. The complete emotion recognition system is demonstrated in Fig. 4. After feature extraction, each speech sample results in statistical values against every emotion: angry, happy, sad, neutral, and disgust. Each emotion in a speech sample has a unique intensity, pitch, zero-crossing rate, and spectral feature. It is important to classify the emotions from the aforementioned feature vectors.

In this study, we have used three classifiers, that is, SVM, k-NN, and RF to train and test our Urdu speech emotional dataset. The multi-class problem in the SVM is also solved by using one-against-one and one-against-all SVM strategies (Hassan & Damper, 2010). These heuristic methods are used to split a multi-class classification problem into multiple binary classification datasets and train a binary classification model on each. The performance of one-against-rest SVM is measured as an average of all binary classifier accuracies. The Urdu speech database is divided into two sets, the training and testing sets, where the training set contains 70% and the testing set contains 30% of the whole dataset. Both sets (training and testing) carry information of each speaker’s emotion. During the model training, feature vectors of the training set along with their labels were given to the classifier whereas in testing, the feature vector of the unclassified sample is given to the model. The performance of classifiers was measured on the test data using accuracy, precision, and recall measures.

Finally, the performance of each classifier was compared for each emotion. Our Urdu speech dataset contains five utterances that are simulated in five different emotions *i.e.*, happy, sad, angry, neutral, and disgust. It was observed that ‘disgust’ is difficult to recognize as compared to the others. It had adverse effects on classification accuracy, while the physiologist also struggled to recognize the disgust emotion. Thus, we divided our data set into two subsets, one with disgust and another without disgust emotion. The classification was implemented in six different ways *i.e.*, females, males, and a complete dataset is subdivided into with and without disgust emotion. In the classification, the emotions angry are labeled as “A”, disgust as “D”, happy as “H”, neutral as “N”, and sad as “S”.

**Figure 5:**
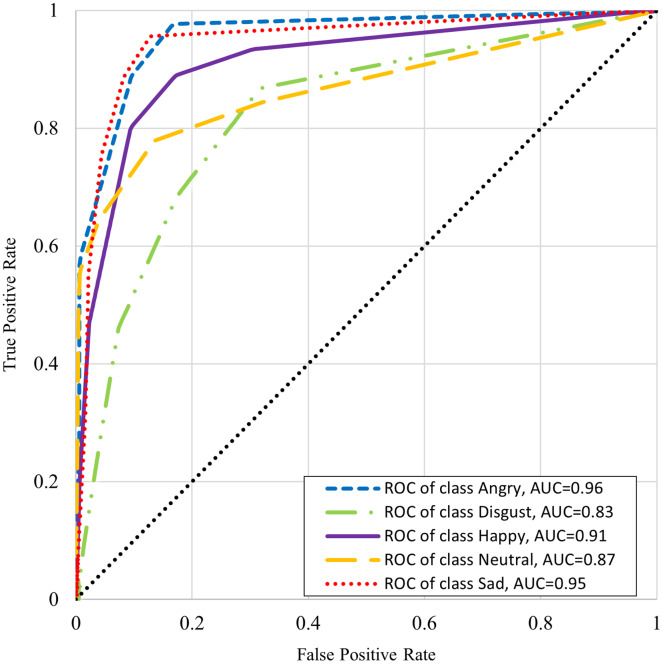
ROC curve of K-NN with disgust emotion.

**Figure 6:**
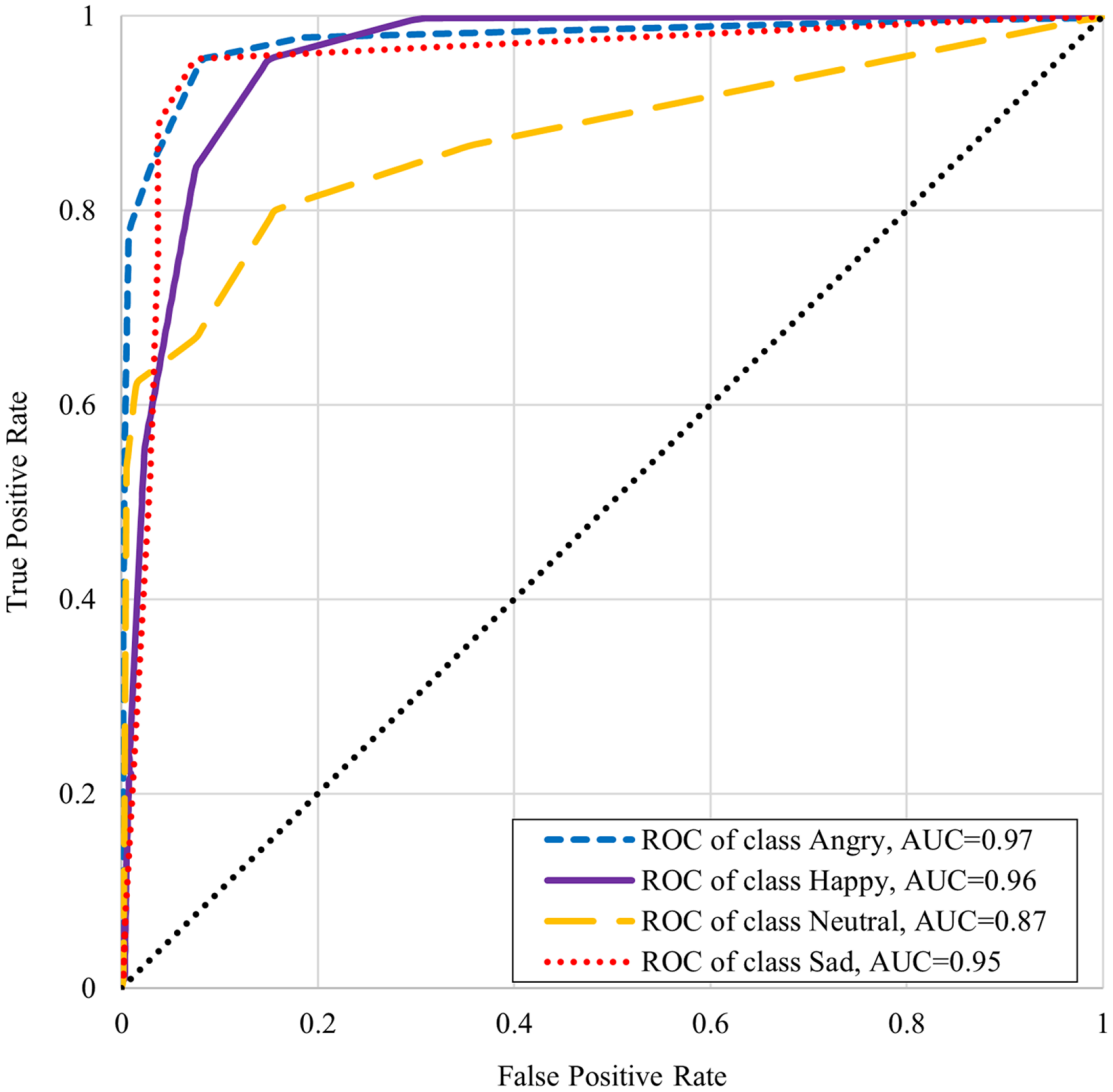
ROC curve of K-NN without disgust emotion.

Table 6 shows the classifiers performance summary with disgust emotion where it can be seen that the k-NN performs better for male and complete datasets. One-*vs*-rest classifier performance is better in the case of the female dataset. Table 7 shows the classifiers performance without disgust emotion dataset. It can be observed that the k-NN performs the best for the male and complete dataset here too, whereas onevs- rest classifier performs better in the case of the female dataset in this scenario. The comparison with state-of-the art from literature is presented in Table 8. It is worthwhile to mention here that although one of the benchmarked studies has reported slightly higher accuracy, our work’s scope is wide in terms of the number of emotions (with five emotions as compared to four emotions) and the size of the dataset (2,500 samples as compared to 400 samples). The receiver operating characteristic (ROC) curve differentiates between the true positive rate or truly classified samples in opposition to the false positive rate or not truly classified samples. A good classification technique has an upside-down “L” shape curve while others follow diagonals. Figures 5 and 6 show the ROC and area under the curve (AUC) for every emotional state *i.e*. angry, happy, disgust, neutral, and sad. These graphs show that AUC of disgust emotion is less as compared to the rest of emotions. Figure 6 shows that the AUCs of the dataset without disgust emotion are much improved as compared to a dataset with disgust emotion. It is concluded that disgust emotion is difficult to recognize than the rest of the emotions.

**Table 6:** Comparison of performance of classification algorithms on emotional speech *corpus* with disgust emotions.

ML techniques	For male only			For female only			Complete dataset		
	Accuracy	Precision	Recall	Accuracy	Precision	Recall	Accuracy	Precision	Recall
One-*vs*-rest	69.5%	71%	69%	68.4%	71%	68%	60.6%	62%	61%
One-*vs*-one	70%	71%	70%	65.6%	67%	66%	62.2%	64%	62%
k-NN	73%	73%	72%	66.4%	69%	66%	66.2%	67%	66%
Random Forest	66.5%	67%	66%	58.8%	62%	59%	60.8%	64%	61%

**Table 7:** Comparison of performance of classification algorithms on emotional speech *corpus* without disgust emotions.

ML techniques	For male only			For female only			Complete dataset		
	Accuracy	Precision	Recall	Accuracy	Precision	Recall	Accuracy	Precision	Recall
One-*vs*-rest	75%	75%	74%	78.5%	81%	79%	70.2%	72%	70%
One-*vs*-one	79.5%	80%	79%	77.5%	78%	79%	70.7%	72%	71%
k-NN	82.5%	84%	83%	76%	76%	76%	76.5%	77%	77%
Random Forest	74%	74%	73%	71%	72%	71%	71.5%	73%	71%

**Table 8:** Comparison with related work.

Papers	Languages	Training technique	Features extraction techniques	Emotions	Classifier used	Accuracy
Tripathi & Beigi (2018)	English and German	Speaker dependent	RNN	Anger, happiness, neutral and sadness	RNN with three layers	71.04%
Kaminska, Sapinski & Anbarjafari (2017)	Polish	Speaker dependent independent	MFCC, BFCC, RASTA, energy, formants, LPC and HFCC	Sadness, happiness, anger, neutral, joy, fear and surprise	SVM and k-NN	81%
Rajisha, Sunija & Riyas (2016)	Malayalam	Speaker dependent	MFCC, STE and pitch	Neutral, anger, happiness and sad	ANN and SVM	78%
Ali et al. (2013)	Urdu	Speaker dependent	Duration, intensity, pitch and formants	Anger, sadness, happiness and comfort	Neive Bayes	76%
Abbas, Zehra & Arif (2013)	Urdu	Speaker dependent	Intensity, pitch and formants	Anger, sadness, happiness and comfort	SMO, MLP, J48 and Neive Bayes	75%
Latif et al. (2018)	Urdu	Speaker independent	LLDs low level descriptor	Happiness, sadness, anger and neutral	SVM, logistic regression and RF	83%
Sinith et al. (2015)	English Malayalam and	Speaker dependent	MFCC, pitch and energy	Anger, neutral sadness and happiness	SVM	70%
Our work	Urdu (with disgust emotion)	Speaker dependent	MFCC, LPC, energy, pitch, zero crossing, spectral flux spectral centroid, spectral roll off	Anger, disgust, happiness, sadness and neutral	k-Nearest Neighbours	73%
Our work	Urdu (without disgust emotion)	Speaker dependent	MFCC, LPC, energy, pitch, zero crossing, spectral flux spectral centroid, spectral roll off	Anger, happiness, sadness and neutral	k-Nearest Neighbors	82 .5%

**Figure 7:**
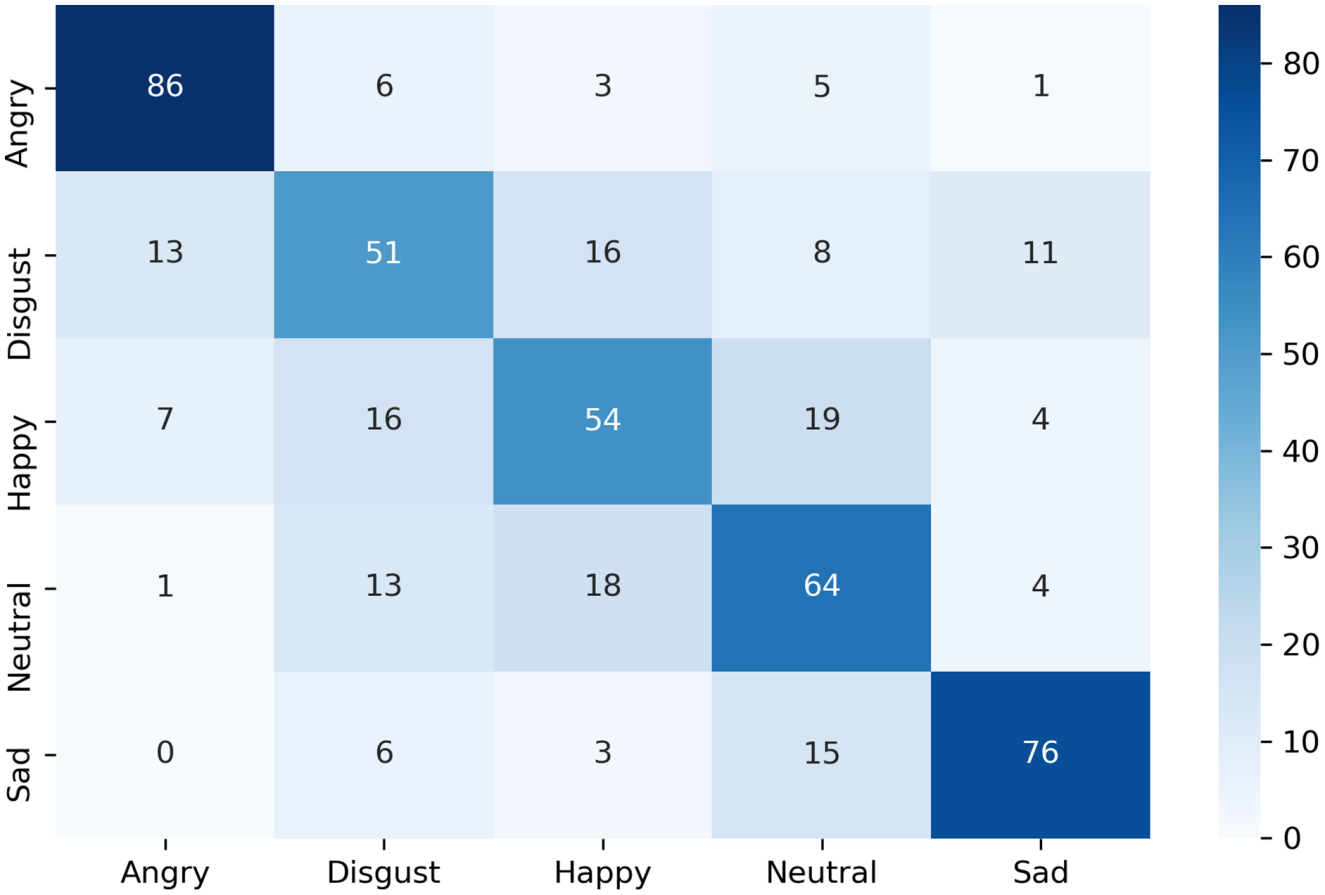
Confusion matrix of k-NN for complete dataset including disgust.

**Figure 8:**
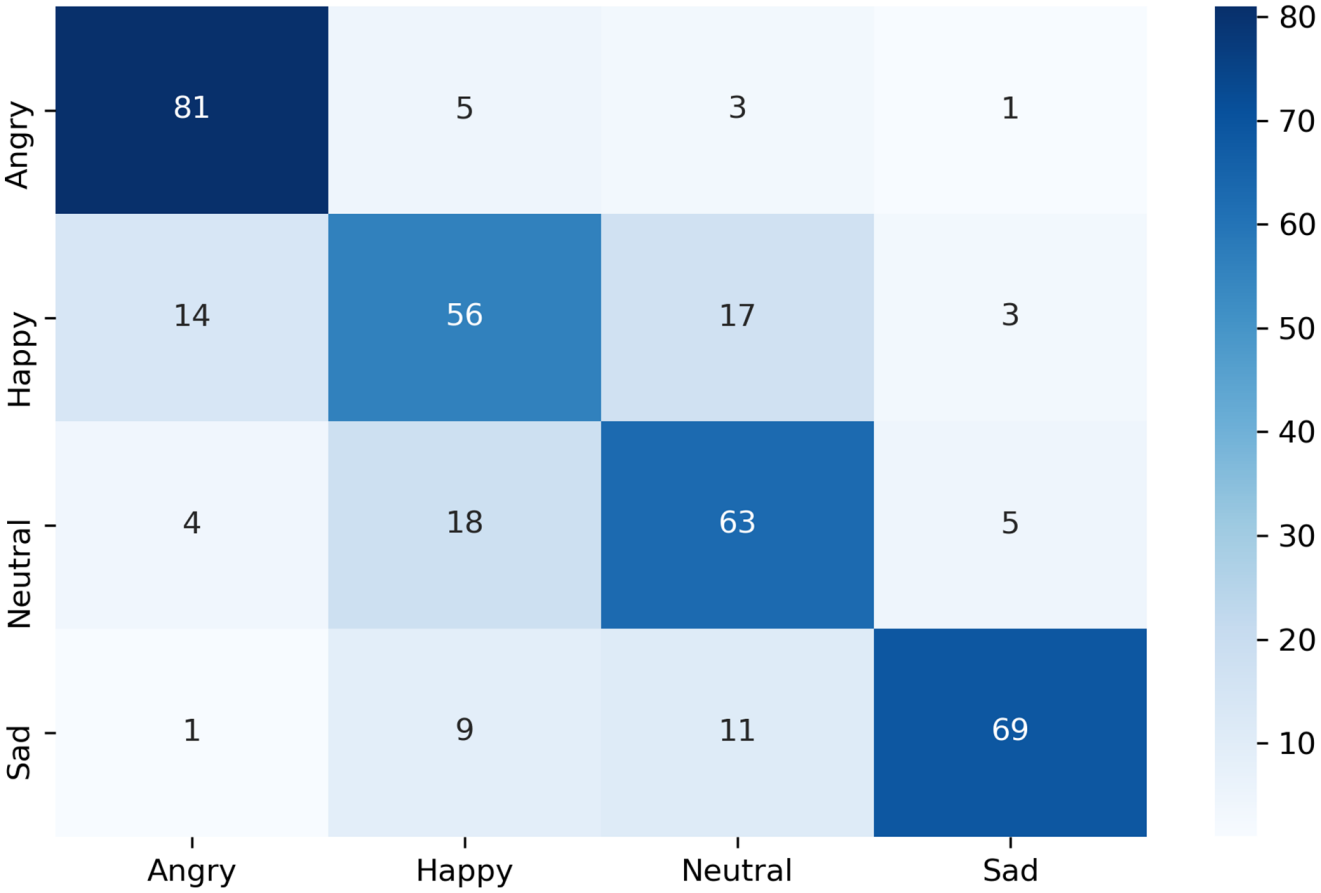
Confusion matrix of k-NN for complete dataset without disgust.

The confusion matrix of the complete dataset with and without disgust emotion is shown in Figs. 7 and 8 respectively, where actual and predicted emotions are listed on vertical and horizontal axis, respectively. As can be seen from Fig. 7, the disgust emotion is the most wrongly predicted class which results in reduction of system accuracy. The confusion matrix without the disgust emotion in Fig. 8 shows a reduction in misclassification of the emotion which thereby results in enhanced accuracy of the system.

## Conclusion

This study presented the design and development of emotional speech *corpus* for the Urdu language. For the development of this *corpus*, five sentences in the Urdu language were simulated in five different emotions, that is, happy, sad, angry, disgust, and neutral. The recognition of emotions from Urdu speech signals using different machine learning techniques was carried out. The Urdu emotional speech data of opposite genders obtains different recognition rates. Different feature sets were studied for better classification of emotions, and only those features were adopted that show a good description of the speech signals. The experimental results showed that males have distinct emotions as compared to the female emotions. There was a large difference in the model performance with disgust and without disgust emotion. The maximum overall recognition accuracy achieved with disgust emotion was 72.5% with k-NN, 68.5% with one-against-rest classifier, and 66.2% on k-NN for male, female, and the complete dataset, respectively. For the dataset without disgust emotion, maximum overall recognition accuracy was 82.5% with k-NN, 78.5% with one-against-rest classifier, and the 76.5% on k-NN for male, female, and the complete dataset respectively.

This study could potentially play a vital role in the automatic human behavior analysis for Urdu speakers. Some of the use cases of the proposed study in human behavior analysis are assessing candidates’ suitability for a job, assessing emotional intelligence, lie detection, etc. In future, we are devoted to developing a more robust Urdu dataset with more emotions and human behaviors.

## Supplemental Information

10.7717/peerj-cs.954/supp-1 Supplemental Information 1 Urdu Emotional Speech dataset. The complete dataset with separate files for the male dataset and the female dataset.

## Additional Information and Declarations

### Data Availability

The following information was supplied regarding data availability: The raw data are available in the Supplemental Files.
